# Insulin, Aging, and the Brain: Mechanisms and Implications

**DOI:** 10.3389/fendo.2015.00013

**Published:** 2015-02-06

**Authors:** Abimbola A. Akintola, Diana van Heemst

**Affiliations:** ^1^Department of Gerontology and Geriatrics, Leiden University Medical Center, Leiden, Netherlands

**Keywords:** insulin, insulin receptors, brain, inflammation, delayed aging, accelerated aging, longevity

## Abstract

There is now an impressive body of literature implicating insulin and insulin signaling in successful aging and longevity. New information from *in vivo* and *in vitro* studies concerning insulin and insulin receptors has extended our understanding of the physiological role of insulin in the brain. However, the relevance of these to aging and longevity remains to be elucidated. Here, we review advances in our understanding of the physiological role of insulin in the brain, how insulin gets into the brain, and its relevance to aging and longevity. Furthermore, we examine possible future therapeutic applications and implications of insulin in the context of available models of delayed and accelerated aging.

## Introduction

Pathways that orchestrate the responses of the organism to changes in its environment have been implicated in the genetic regulation of lifespan across different species. One of the key pathways identified by genetic analysis of long-lived *Caenorhabditis elegans* (*C. elegans*) mutants is insulin/insulin-like growth factor-1 (IGF-1) signaling (IIS) (1, 2). In invertebrates, multiple insulin/IGF-1-like ligands signal via a common receptor, which shows homology to the mammalian insulin and IGF-1 receptors. Also in mammals, insulin/IGF-1 signaling has been linked to aging, lifespan, and longevity (3). Although in mammals, insulin and IGF-1 act predominantly via distinct receptors, there is extensive overlap and interaction in their downstream signaling cascades, making it difficult to separate effects of insulin signaling from those of IGF-1 signaling. The long-lived phenotype of FIRKO mice, which were made by selective disruption of the insulin receptor (IR) in adipose tissue, supports a role of insulin signaling in longevity (4). Moreover, many of the long-lived mouse mutants with disrupted GH/IGF-1 signaling display enhanced insulin sensitivity. In humans, a hallmark phenotype of healthy longevity is maintenance of insulin sensitivity (5, 6), which has been observed in familial human longevity (7, 8), as well as in centenarians (9–11). Insulin influences all aspects of human physiology (12, 13). Besides regulating peripheral glucose homeostasis, insulin is an important neuromodulator that contributes to neurobiological processes (14), with growing evidence that insulin supports behavioral, cellular, biochemical, and molecular functions (15). In literature, evidence linking aging and insulin signaling includes prolongation of life span in rodents via genetic mutations affecting insulin signaling pathways or via interventions that down-regulate nutrient sensing pathways such as caloric restriction. Further evidence includes data on the role of type 2 diabetes in accelerated aging syndromes, and the increased incidence of insulin resistance with age (16). In model organisms (nematodes and fruit flies), specific neural manipulations of insulin signaling have also been linked to aging and lifespan (17, 18). Insulin is produced in the brain of these organisms, making it undoubtedly a neuropeptide. In mammals and humans, IRs are highly abundant in many brain areas and nuclei, but it remains unclear if insulin is produced in the brain. Furthermore, the physiological and pathophysiological mechanisms of insulin action in the brain in relation to aging and longevity remain to be elucidated.

With the global population aging, there has been an astonishing increase in the prevalence of obesity (19), metabolic syndrome (20), type 2 diabetes (21), and neurodegenerative diseases (22). Insulin resistance is a shared feature in these diverse pathologies (13, 23–26). It therefore becomes critical to understand the role of insulin in healthy longevity, as this may be relevant to combatting age-related disorders that have been linked to disturbances in glucose metabolism. The aim of this article is to review advances in our knowledge about insulin, insulin signaling, and the brain, and to present these in the context of available models of delayed and accelerated aging. Furthermore, we will examine the links between inflammation, metabolic health, and brain health, and their effect on aging. Finally, we will review therapeutic options to enhance brain insulin action, including measures to enhance local brain insulin levels as well as measures to enhance the brain responses to insulin.

## Insulin and the Brain: A Century of Discoveries

Insulin, after discovery in 1921, was initially considered a peripheral hormone and thus unable to cross the blood–brain barrier (BBB) (27). However, in 1967, Margolis and Altszuler demonstrated in dogs that the concentration of cerebrospinal fluid (CSF) insulin increased after an increase in plasma insulin (28), thus showing that insulin is able to cross the blood–CSF barrier. In 1978, Havrankova et al. demonstrated the widespread presence of IRs in the central nervous system (CNS) of the rat (29). Later that year, they further demonstrated that high levels of insulin were present in rat brain extracts, and found that the concentration of insulin in the CNS was considerably higher than its concentration in the circulation (30). They thus proposed a physiological role for insulin in the CNS. In the 1980s, further evidence that insulin from peripheral circulation crosses the BBB thus gaining access to the brain was provided. In 1983, Dorn et al. demonstrated that the human brain contains insulin in concentrations much higher than the blood, the highest being in the hypothalamus (31). Furthermore, they showed the presence of high concentrations of insulin in the brains and spinal cords of human cadavers, mice, and rats (32). Baskin et al. demonstrated uptake in the hypothalamus of [125I]iodoinsulin after the insulin had been stereotaxically injected into a lateral cerebral ventricle. Furthermore, they detected insulin-like immunoreactivity in the periventricular, supraoptic, suprachiasmatic, arcuate, and lateral hypothalamic nuclei of the rat hypothalamus (33, 34). In 1992, Schechter et al. delineated the ontogeny of rabbit brain insulin concentration and demonstrated that insulin availability is developmentally regulated (35). In the past decade, studies of the effects of insulin in the brain have been enhanced after development of non-invasive methods of selective delivery of insulin into the brain, via the intranasal route, circumventing peripheral effects of systemic hypoglycemia (36). This has advanced our understanding of potentially therapeutic effects of enhancing insulin concentrations in the brain. Furthermore, studies in recent years have brought forward the role of insulin signaling in the hypothalamus, as a key player in regulation of hepatic glucose production and food intake (37).

### Brain insulin: is insulin a neuropeptide in humans?

**Figure 1:**
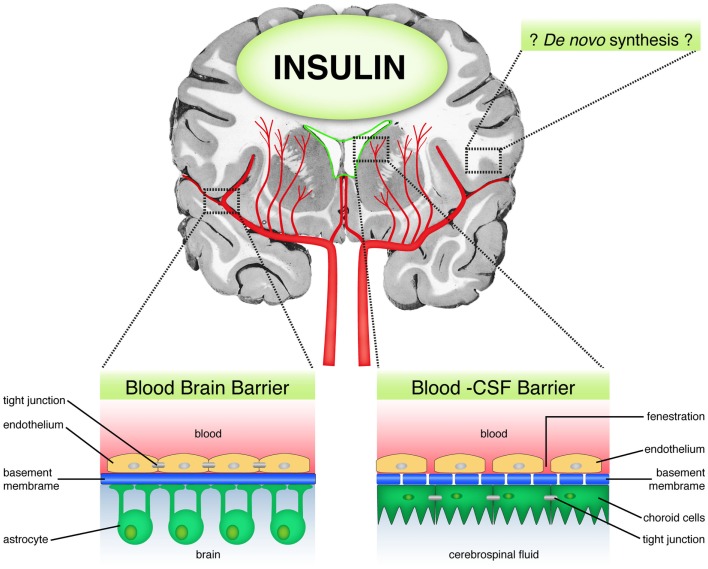
**Sources of brain insulin**. Schematic diagram showing the possible sources of brain insulin. First, peripheral insulin can access the brain through the blood brain barrier (BBB) via a selective, carrier-mediated transport system. Second, insulin may diffuse through the blood–CSF barrier in circumventricular regions, which are lacking in BBB. Third, there is some limited evidence suggesting the possibility of *de novo* insulin synthesis in the brain.

In the rabbit, discordance was observed between insulin concentrations in serum and CSF (35). Insulin was found to be present in high concentrations in brain micro-vessels (38), brain extracts (39), and immature nerve cell bodies (35, 40), despite that only 0.046% of peripheral insulin crosses the BBB in mice (12, 41). Moreover, brain insulin concentrations were observed to vary according to developmental stages, with peak amounts being observed during the critical phases of brain growth and development (42). Taken together, these results suggest that brain insulin availability is strictly regulated and can reach high levels in the CNS. This raises the question as to the source of brain insulin, does all of brain insulin derive from the periphery or is insulin also synthesized in the brain (Figure 1)?

There is unequivocal evidence for selective, regulated, time dependent, temperature sensitive, carrier mediated, and saturable insulin transport to the brain (43–50). In mice, human insulin was shown to access the CNS after crossing the BBB (51). In rabbits, insulin infused into the carotid artery was shown to have crossed the BBB into the peri-capillary space and brain parenchyma with preservation of the peptide’s integrity (45). In dogs, studies using a three component mathematical model (plasma, intermediate component, and CSF) have shown that insulin delivery to the CNS fits a receptor-mediated saturable process (43). In healthy humans, during hyperinsulinemic, euglycemic clamp studies, increase in circulating insulin was demonstrated to rapidly affect brain activity, alongside rapid cerebral insulin signal transduction, independent of the systemic effects of the insulin (48).

Apart from passage through the BBB, direct access of insulin to the CSF has also been demonstrated (Figure 1). This alternative route occurs through circumventricular regions, such as the area postrema, which lack a BBB (34, 52–54). Unlike the BBB that contains tight junctions, the capillaries in the circumventricular regions are porous, thereby allowing plasma solubles to diffuse freely and directly into these areas (55). The route through which insulin accesses the brain has implications for the rate of convection and diffusion in the brain, and distribution of the insulin into the brain parenchyma. Following intraventricular administration of insulin, insulin becomes distributed through the ventricular compartments and to the surface of the brain bathed by the subarachnoid space, with relatively slow rate of diffusion into the brain parenchyma, and is minimal at distances more than 1–2 mm removed from the CSF surface (55, 56). In addition, insulin delivered into the CSF undergoes relatively rapid bulk flow through the CSF flow tracks. For example, the entire CSF volume is turned over every 4–5 h following production at the choroid plexus in the human brain (55).

In model organisms, insulin is biosynthesized by neurons in the brain and it exerts both local and remote actions, including regulation of homeostasis; making it undoubtedly a neuropeptide. In humans, however, insulin is mainly produced in the pancreas, which raises the question as to whether insulin can be considered a neuropeptide in humans. Neuropeptides have been defined as “small proteinaceous substances produced and released by neurons through the regulated secretory route and acting on neural substrates” (57). Neuropeptides have been shown to have strict, cell specific expression patterns, on which the physiological or behavioral role of the peptides is based. Criteria for classification as a neuropeptide include gene expression and biosynthesis by neurons; storage, and regulated release upon demand and ability to modulate or mediate neural functioning directly through receptors (57). Although IRs are highly abundant in many brain areas and nuclei, it remains unclear if insulin is produced in the brain. Therefore, following the strict criteria for neuropeptide definition, it becomes debatable if mammalian insulin is a true neuropeptide.

Evidence in favor of insulin synthesis in the brain mostly derives from *in vitro* studies, including the study by Clarke et al. in 1986, which demonstrated the synthesis of insulin by cultured rat brain neuronal and astrocyte glial cells and their release of insulin in primary culture. The insulin release after membrane depolarization of the neurons was biphasic, in a manner similar to that of pancreatic beta cells (58). In 1990s, Schechter et al. provided both *in vivo* and *in vitro* evidence from mammalian brains supporting the *de novo* synthesis of insulin. *In vitro* evidence included the demonstration of preproinsulin I and II mRNA in neuron cell cultures of fetal rat brains (59). From *in vivo* studies, presence of preproinsulin I and II mRNAs and insulin immunoreaction was detected within the rough endoplasmic reticulum, the Golgi apparatus, cytoplasm, axon, synapsis, and dendrites of the rat fetal brain (40).

Summarily, as can be seen in Figure 1, whether insulin is derived from the periphery, local sources or both, insulin is present in the CNS, where it subserves many functions and contributes to neurobiological processes.

### Activation of insulin receptors in the brain

**Figure 2:**
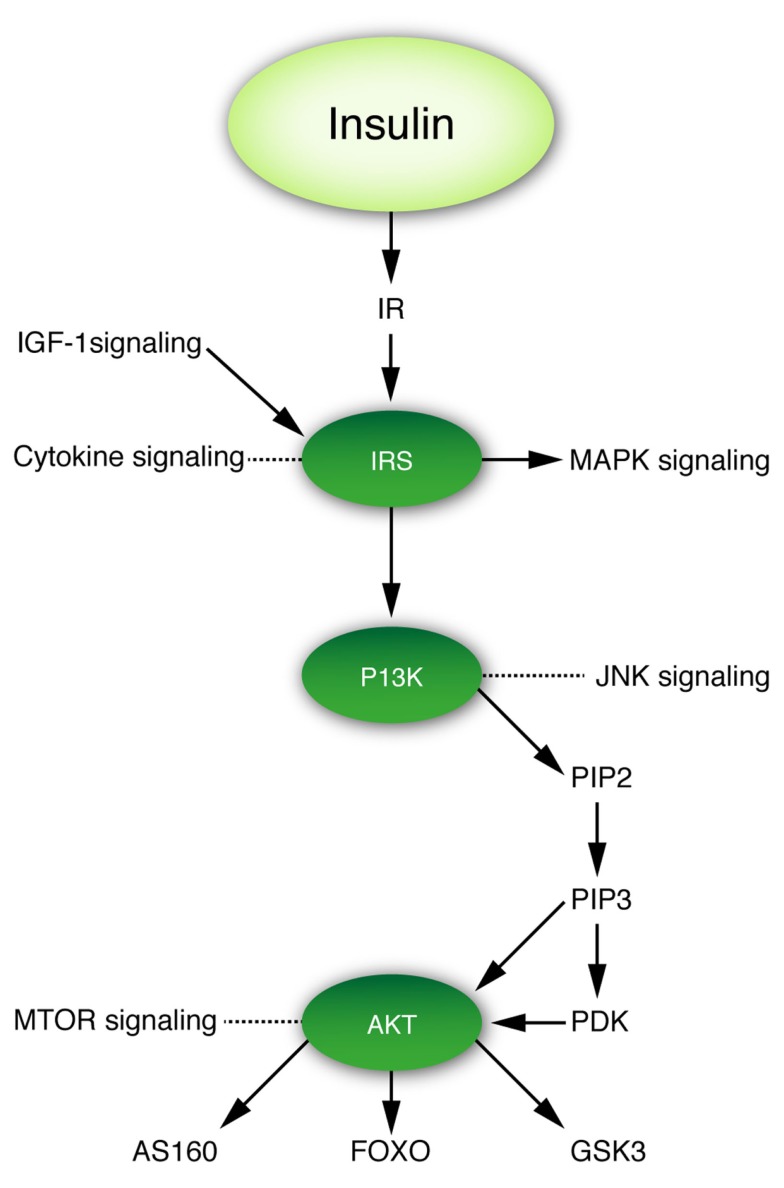
**Insulin–IRS–P13K–AKT signaling cascade and its crosstalk with other signaling pathways**. Figure denotes three critical nodes in insulin signaling that are important for the interaction of insulin signaling with other pathways relevant to this review.

As in peripheral tissues, insulin signaling in the brain occurs mainly via the IR pathway (Figure 2), which contains several critical nodes of interaction with other signaling pathways (60). Activation of the insulin signaling cascade starts with binding of the insulin ligand to the IR, which belongs to the family of tyrosine kinase receptors, auto phosphorylation of which is essential for their activation. Upon activation, the IR phosphorylates insulin receptor substrate (IRS) proteins. IRS proteins are also activated upon binding of the IGF-1 ligand to its cognate receptor. Thus, IRS proteins represent a critical node of conversion of the insulin and IGF-1 signaling cascades, and their crosstalk with other pathways, such as cytokine signaling. In addition to its activation of the Ras–mitogen-activated protein kinase (MAPK) pathway, activated IRS proteins serve as docking sites for the assembly and activation of, among others, phospho-inositol-3 kinase (PI3K), which generates the lipid second messenger phosphatidylinositol 3,4,5-triphosphate (PIP3). PI3K represents another critical node of crosstalk with other signaling pathways, including the c-Jun-N-terminal kinase (JNK) stress signaling pathway. Elevated levels of PIP3 activate phosphoinositide-dependent protein kinase-1 (PDK1) and AKT. AKT represents yet another critical node of interaction with the mammalian target of rapamycin (mTOR) nutrient signaling pathway. AKT targets include glycogen synthase kinase 3 (GSK3), Akt substrate of 160 kDa (AS160, phosphorylation of which is required for translocation of the glucose transporter GLUT4 to the plasma membrane) and forkhead transcription factors (FOXOs) (Figure 2). Phosphorylation of FOXOs induces their translocation from the nucleus, which causes profound changes in the transcription of key factors implicated in metabolism, cell cycle regulation, apoptosis, and resistance to oxidative stress (61).

### Distribution of insulin receptors in the brain

In higher mammals and humans, IRs are widely distributed throughout peripheral tissues, with their main function being to transport glucose into cells, inhibit glucose production and increase glucose uptake by triggering signaling pathways in the liver, muscle, and fat (62). The IR consists of a tetramer, with two alpha subunits and two beta subunits. Brain IR subunits differ structurally from peripheral IR subunits in that they have a lower molecular weight (63) and can withstand exposure to high concentrations of insulin without undergoing down-regulation (64, 65). The mammalian brain has specific IRs (29, 66), which are of two types. One is the neuronal/neuron-specific type, which is abundant in the neuron (67), while the second type is the non-neuronal/peripheral-like type, with lower density in glial cells (38, 66, 68). Insulin receptors are highly abundant in the neurons, with high protein concentrations in cell bodies and synapses, and less abundant in the glia. Brain IRs are abundant in the brain, but are highly enriched in the olfactory bulb, hypothalamus, hippocampus, cerebellum, amygdala, and cerebral cortex (12).

Growing but controversial evidence suggests that the specific regional concentrations of IR reflect different IR functions associated with particular brain regions. IR enrichment in the hypothalamus and limbic system including the hippocampus, pyriform cortex, and amygdala, areas that reciprocally connect and communicate with each other, has been proposed to be suggestive of a role in emotion and higher cognitive functions, particularly learning and memory (69, 70). Higher IR concentrations are found in the hippocampus, which is critically involved in spatial memory processing, suggesting insulin’s role in learning and declarative memory (69). Evidence that synthesis of IR may be increased in these hippocampal areas as a result of learning is supported by the up-regulation and the changes in distribution patterns of IR mRNA in the hippocampus and dentate gyrus following water maze training in rats (70). Insulin is involved in the regulation of food intake, which is consistent with the high concentrations of IR in the olfactory bulb and the hypothalamus. Furthermore, the high concentration of IR in the choroid plexus suggests that it may be required for transport of peripheral insulin across the blood–CSF barrier (70).

### Functional significance of insulin in the brain

As the most potent anabolic hormone yet identified, insulin has both metabolic and non-metabolic functions. Insulin regulates food intake, as well as glucose, lipid, and energy homeostasis and stimulates synthesis (as well as inhibition of breakdown) of glycogen, triglycerides, and most proteins. It is also involved in regulation of hedonic behavior and non-homeostatic control of intake of food and other substances via reward processing.

### Non-metabolic functions

The presence of insulin and IRs in the brain indicates that the brain is a target organ for insulin. Insulin plays a key role in synaptic plasticity, apoptosis, mood, learning, reproduction, and growth (37, 71–74). Insulin and IR expression in the brain has been suggested to exert neurotrophic effects on CNS neurons (75). Insulin has been considered to support neuronal protein synthesis and cytoskeletal protein expression (75), neurite outgrowth (76, 77), migration, and differentiation in the absence of other growth factors (78, 79), and nascent synapse formation (75, 80). It promotes growth and regeneration of axonal sprouts, especially small sized sensory neurons (81), neuronal survival, circuit development, synaptic plasticity (82), and postsynaptic neurotransmitter receptor trafficking (80). Evidence in favor of insulin’s role as a neurotransmitter in the CNS includes the observations that (i) insulin is present in neurons (67), (ii) neurons contain specific IRs (64), and (iii) insulin affects neuronal firing and catecholamine metabolism (83–85). Insulin also has effects on BBB function, including ability to affect the transport of other substances. Binding sites for insulin have been described at both the choroid plexus and on brain endothelial cells (86, 87). Insulin also has neuro-protective properties (88–90). Central insulin plays a role in cognitive processes such as attention, executive functioning, learning, and memory (91), and direct application of insulin to the CNS in humans has been shown to improve memory and cognition (92, 93). Thus, insulin is involved in attributes that are essential for healthy aging.

### Metabolic functions

The brain plays a key role in maintenance of homeostasis, or the ability to maintain vital parameters of the internal environment within narrow limits, despite fluctuations in the external environment. Metabolic homeostasis requires the integration of numerous cues reflecting energy availability by the hypothalamus and nearby brain structures, to mount a coordinated response to adapt fuel flux so as to maintain energy homeostasis. Insulin is one of the many cues informing the brain about energy status. Research on insulin signaling has primarily focused on insulin-mediated processes in the classical insulin target organs. These include glucose uptake into skeletal muscle, inhibition of glucose production by the liver, and inhibition of lipolysis in adipose tissue. However, in 1979, a role for insulin in the central regulation of energy homeostasis was suggested based on the observations that insulin levels circulate in proportion to fat mass in most mammals and that intra-cerebroventricular insulin administration results in a dose dependent reduction in food intake and body weight in monkeys (94). In line, IRs are expressed throughout the mammalian brain (29). Metabolic syndrome and diabetes have traditionally been considered as peripheral metabolic diseases. Recently, various non-invasive brain-imaging techniques have revealed structural and functional abnormalities that are associated with diabetes. Critical autonomic regulatory neurons in the hypothalamus and brainstem are responsible for maintenance of energy homeostasis and functional changes in these areas are associated with the development of diabetes (95). It was also shown that after hepatic branch vagotomy the suppression of hepatic gluconeogenesis induced by increasing circulating insulin levels was reduced by half (96). Mechanistically, binding of insulin to the IR and activation of the PI3K pathway in hypothalamic glucose-responsive neurons, which was shown to induce their hyperpolarization by opening of ATP-dependent potassium channels (97), has been implicated in the central effects of insulin on hepatic glucose production (96). Recently, it was shown that ingestion of a glucose solution resulted in a prolonged and significant blood oxygen dependent decrease in activity in the hypothalamus of healthy subjects, but not in type 2 diabetic patients (98). Insulin is also involved in regulation of energy homeostasis via IR in the ventromedial hypothalamus and acts on the brain to suppress feeding (99). Thus, insulin acts as a satiety factor, a finding supported by the observation that the response of glucose-excited neurons in the ventrolateral and ventromedial hypothalamic nucleus to decreased glucose is blunted by insulin (100).

Taken together, these data indicate that, beside peripheral insulin resistance, reduced brain insulin action may also contribute to loss of maintenance of metabolic control. Indeed, brain specific deletion of the IR was shown to result in enhanced food intake in female mice; and in mild obesity, hyperleptinemia, insulin resistance, and hypertriglyceridemia in both male and female mice (101). In line with these findings, in rats, decreasing hypothalamic IRs caused overeating and insulin resistance and hypothalamic insulin signaling was shown to be required for inhibition of glucose production (102). High-fat diet-induced obesity is associated with reduced brain insulin transport and an impairment of insulin action when given directly into the CNS, suggesting a loss of the effectiveness of insulin in the CNS to provide feedback signaling in circumstances of chronic hyperinsulinemia (103). Upon aging, peripheral insulin resistance progressively increases, inducing compensatory chronic elevations in circulating insulin levels. Therefore, central insulin action will be discussed in the context of models of delayed and accelerated aging.

## Insulin and the Brain: Models of Delayed Aging

### Nematode models of delayed aging

There is an impressive body of literature implicating insulin/IGF-1 like ligands and insulin/IGF-1 signaling in the regulation of metabolism, development, and longevity in the roundworm *C. elegans* (104). In response to unfavorable stressful environmental conditions, *C. elegans* larvae can transiently exit the cycle of growth and development to sexual maturity by transformation into developmentally arrested, non-feeding, stress resistant, and long-lived dauer larvae (105, 106). It was found that several dauer formation defective (daf) mutants are also long-lived, possibly because these mutants display specific key features of the dauer stage while developing in sexually mature adults, such as enhanced resistance to multiple stresses due to induction of cytoprotective pathways (107). Of the many long-lived daf mutants in nematodes, the ones that are best characterized comprise the *daf-2*, *age-1 (daf 23)*, *daf-16*, and *daf-18* mutants. Cloning and sequencing of the loci affected in long-lived daf mutants has revealed that these show strong sequence homology with evolutionarily conserved components of the mammalian insulin/insulin-like growth factor-1 signal transduction cascade (108–110). For example, the *daf-2* gene that has been shown to regulate lifespan in *C. elegans*, and the related tyrosine kinase receptors InR in *Drosophila melanogaster* (*D. melanogaster*) encode components that are homologous to the mammalian insulin and insulin-like growth factor-1 receptors. In response to food or the perception of food, multiple insulin-like ligands are secreted from neurosecretory cells in the brain of *C. elegans* (111) and *D. melanogaster* (112), indicating that in these invertebrates, the CNS plays a key role in insulin signaling mediated regulation of physiology and lifespan in response to environmental cues. Moreover, more than 10 years ago, Wolkow et al. (17) demonstrated that restoration of the daf-2 pathway of insulin-like signaling in neurons alone was sufficient to restore wildtype lifespan in *C. elegans*, and thus provided further evidence as to the role of insulin in the nervous system as a central regulator of animal longevity.

### Mouse models of delayed aging

In mammals, the insulin/insulin-like growth factor-1 signaling cascade exhibits some striking differences compared to the insulin/insulin-like growth factor-1 signaling cascade in invertebrates (113). These differences include the acquisition of GH as a main regulator of IGF-1 production by the liver, and the acquisition of separate receptors for insulin and IGF-1. Again, several of the existing long-lived mammalian mutants with defects in insulin/IGF-1 signaling point to a role of the CNS in the regulation of mammalian longevity. The mutations that have thus far been most consistently and most strongly associated with increases in lifespan in mice comprise the *Prop-1* mutation displayed by Ames dwarf mice (114) and the *Pit-1* mutation displayed by Snell dwarf mice (115). These two mutations confer a defect in the development of the anterior pituitary gland, which causes a lifelong combined hormonal deficiency in growth hormone, thyroid stimulating hormone, and prolactin. In these as well as other long-lived mice, longevity has been strongly correlated with enhanced insulin sensitivity (116). In addition to the Ames and Snell dwarf mice, many other mouse mutants with defects in insulin/IGF-1 signaling have been described to display a longevity phenotype, which strongly implicates the insulin/IGF-1 signaling pathway in the regulation of rodent longevity. Involvement of both insulin signaling and IGF-1 signaling in mouse longevity was suggested by the long-lived phenotypes displayed by mice with selective disruption of the IR in adipose tissue (4) and mice heterozygous for mutation of IGF-1R (1). Summarily, improved insulin control (of carbohydrate homeostasis) has been identified as one of the pathways implicated in the remarkable extension of longevity in long-lived mouse mutants (117).

### Human models of delayed aging

**Figure 3:**
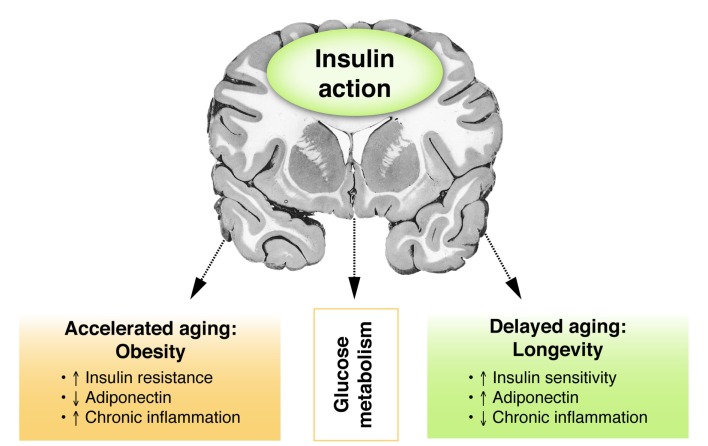
**Insulin and the brain: models of accelerated and delayed aging**. Figure showing the putative relationship between central insulin action and glucose metabolism in models of accelerated or delayed aging. Obesity as a model for accelerated aging is associated with peripheral insulin resistance, decreased adiponectin levels, and enhanced chronic inflammation. Opposite features are observed in healthy longevity as a model of delayed aging.

Also in humans, preserved insulin sensitivity has been associated with longevity. Insulin resistance has been shown to predict the development of age-related diseases, including hypertension, coronary heart disease, stroke, cancer, and type 2 diabetes (118). In the general population, the association between aging and decline in insulin sensitivity (119–123) has been demonstrated in several studies (Figure 3). Mechanisms suggested to contribute to decreased insulin sensitivity in the elderly include (i) age-related receptor and post-receptor defects in insulin action (124, 125), (ii) an age-related decrease in insulin stimulated whole body glucose oxidation (126), (iii) an age-related reduction in beta cell response to glucose (126), and (iv) impaired insulin-mediated glucose uptake, and inability to suppress hepatic glucose output (127, 128). In contrast, centenarians, which exhibit exceptional longevity, seem protected against the age-related decline in insulin sensitivity when compared to a group of advanced middle-aged individuals. (11) Of note, a methodological difficulty that is associated with the comparison of groups that differ in calendar age is potential confounding by the changes that occur in body composition and endocrine function with advancing age. Moreover, differences may exist between different birth cohorts in environmental impacts, including differences in the availability vaccinations or medications (e.g., antibiotics).

The relationship between longevity and preserved insulin action has also been observed in studies of familial longevity. In the Leiden longevity study, offspring of long-lived nonagenarian siblings, having inherited on average 50% of the genetic propensity of their long-lived parent were included together with the partners of the offspring (129), with whom they have shared the same socio-economic and geographical environment for decades and who are of a similar age. We showed that already at middle age the offspring from these long-lived siblings displayed a decreased mortality risk suggesting that there is indeed evidence for genetic enrichment for longevity (129). Moreover, human offspring of exceptionally long-lived siblings, when compared to their partners showed a remarkably lower prevalence of metabolic syndrome (130) and diabetes (131). After exclusion of diabetic patients, the offspring of exceptionally long-lived siblings displayed lower circulating levels of glucose and slightly lower circulating insulin levels (7). Using hyperinsulinemic euglycemic clamps studies, we could show that the offspring of long-lived siblings specifically displayed enhanced peripheral insulin sensitivity compared to age matched controls (8). A study using high field (7-T) MR spectroscopy of the tibialis anterior muscle indicated that the enhanced peripheral insulin sensitivity of offspring is associated with lower intramyocellular lipid content, which may be indicative of better mitochondrial capacity (132).

The mechanisms underlying the preserved insulin action in centenarians as well as in offspring of nonagenarian siblings remain unclear. However, suggested mechanisms include genetic enrichment for favorable features related to body fat and lipoprotein distribution, reduced plasma free radical concentrations, and enhanced cellular response to oxidative stress and immune function (11, 133, 134). Taken together, these results suggest that maintenance of insulin sensitivity is a key feature of healthy longevity.

## Insulin and the Brain: Models of Accelerated Aging

### Obesity as a model for accelerated aging

The most common acquired factors causing insulin resistance are obesity and a sedentary lifestyle. Obesity and the associated increase in body fat are the consequences of chronic, long-term nutrient excess. In Western societies, the prevalence of obesity continues to increase and numerous studies have demonstrated an association between obesity and enhanced mortality risk (135). The relationship between obesity and excess mortality is consistent with evidence that obese individuals are at increased risk of essential hypertension, type 2 diabetes mellitus (DM2), and cardiovascular disease (CVD). It has been suggested that insulin resistance is the major contributor to clinical outcomes associated with obesity (136).

It is not known why some obese individuals develop insulin resistance while others remain insulin sensitive (137). A potential mechanism that might explain the association between excess adiposity and peripheral insulin resistance is impaired adipogenesis and reduced lipogenesis in subcutaneous fat, which would lead to enhanced deposition of fat in the visceral depots and larger sizes of visceral adipocytes (137, 138). Increased visceral adiposity is associated with enhanced secretion of inflammatory cytokines and induction of insulin resistance (139). Nutrient excess results in enhanced exposure of cells and tissues to high levels of circulatory glucose and fatty acids. These exposures can activate various intracellular inflammatory pathways and lead to mitochondrial dysfunction, reactive oxygen species (ROS), ER stress, and the associated unfolded protein response that induce resistance to both leptin and insulin (140). ROS can have both stimulatory and inhibitory effects on insulin signaling. It was shown that under normal physiological conditions, optimal activation of the IR requires redox priming by IR mediated activation of NAD(P)H oxidase (NOX) in many cell types (141). In addition, mild bursts in intracellular ROS can activate the IR receptor independent of insulin, allowing for ROS mediated ligand activation bypass of IR signaling (142). In contrast, increased levels of ROS or prolonged exposure to oxidative stress have been shown to inhibit insulin signaling and to induce insulin resistance (143). Enhanced exposure of skeletal muscle to high levels of fatty acids in circulation can result in enhanced levels of intramyocellular triglyceride storage. Because intramyocellular lipid droplets are stored in close vicinity to mitochondria, which constitute the main intracellular source of ROS, intramyocellular triglycerides are very vulnerable to oxidation. Upon peroxidation of intramyocellular triglycerides toxic lipid species are generated, including diacylglycerol (DAG), ceramide, and long-chain fatty acyl-CoA, which impair insulin signaling (143). Both enhanced influx, as a consequence of nutrient excess, and reduced efflux, as a result reduced oxidative capacity and mitochondrial dysfunction have been implicated in the accumulation of toxic intramyocellular lipids (144, 145). In support of a role of reduced efflux due to mitochondrial dysfunction, non-obese, insulin sensitive first degree relatives of patients with type 2 diabetes were shown to display impaired ability to switch to fat oxidation after high-fat intake (146), as well as higher levels of intramyocellular lipids and reduced oxidative capacity (147). These data implicate ROS and mitochondrial dysfunction in the development of insulin resistance.

It is unknown via which mechanisms insulin resistance is associated with a shortening of lifespan. If peripheral organs, such as skeletal muscle and adipose tissue become less responsive to insulin, euglycemia will be maintained by the capacity of the pancreas to hypersecrete insulin so as to overcome insulin resistance at peripheral organs. Exposure to continuous surges of hyperinsulinemia may overstimulate other tissues that have remained normally responsive to insulin, such as the liver, resulting in a pro-atherogenic lipid profile (148). Other data implicate adiponectin in the association between insulin resistance and lifespan. Adiponectin, an anti-inflammatory adipokine secreted by adipose tissue (149) was found to be negatively correlated with adipocyte size and obesity (150). Interestingly, elevated adiponectin levels have been observed in long-lived mice, such as the Ames dwarf mice (151) as well as in long-lived humans, such as centenarians (152–154). Recently, effects of adiponectin on peripheral insulin sensitivity also implicate central effects on reduction of high-fat diet-induced hypothalamic inflammation and insulin resistance (155).

## Inflammation and the Brain

### Inflammation and aging: inflammaging

Inflammaging is characterized by the increase in chronic, low-grade inflammation in the absence of overt infection that occurs with aging (156). Inflammaging as well as the circulatory markers that characterize this state, including C-reactive protein (CRP), interleukin-6 (IL-6), tumor necrosis factor α (TNF-α), and interleukin 1 beta (IL1beta) are strong risk factors for many age-related diseases and mortality. It is thought that part of these circulatory factors are produced locally, after which these leak into the circulation. Different sources that contribute to the state of inflammaging include the accumulation of cellular debris and organelle components, accumulation of senescent cells, immunosenescence, changes in the gut microbiome and deregulation of the coagulation system.

Macromolecules, cells, and tissues are continuously damaged and repaired. Chronic inflammation is part of regular tissue remodeling as it facilitates tissue repair and turnover. However, a persistent inflammatory response can lead to tissue degeneration by activated leukocytes, cytokines, or collagen deposition. In literature, one key structure where links between inflammation and aging are emerging is the hypothalamus.

### Hypothalamic inflammation

The hypothalamus is the seat of control of various metabolic and non-metabolic processes in the body, and is responsible for maintenance of homeostasis from early life through to aging. Besides its role in the synthesis and secretion of neurohormones, the hypothalamus regulates energy balance, stress responsiveness, as well as lipid and glucose metabolism. Diet-induced obesity has been shown to be associated with central leptin and insulin resistance (157). High-fat feeding has been shown to induce hypothalamic inflammation, which has been linked to the development of insulin resistance and obesity (157–159). In 2005, De Souza et al. demonstrated that 6 weeks of high-fat feeding induced impaired functional and molecular activation of the insulin-signaling pathway, with accompanying expression of several pro-inflammatory cytokines (IL-1β, TNFα, and IL-6) and inflammatory responsive proteins in the hypothalamus (158). Moreover, hypothalamic inflammation was shown to decrease the efficacy of central insulin administration to inhibit lipolysis, even before the onset of peripheral insulin resistance in white adipose tissue (160). Recently, a series of experiments in mice has demonstrated that hypothalamic inflammation occurs rapidly after high-fat feeding and is mediated by hyper activation of hypothalamic microglia, which was associated with gliosis in the ARC nucleus and eventual reduction in the number of POMC neurons, which are key in the regulation of energy homeostasis and adiposity (161).

Microglia are resident macrophages that play an important role in the clearance of cell debris via phagocytosis and the release of pro-inflammatory cytokines to recruit other immune responsive cells to the sites of injury in the CNS, including blood-borne macrophages. It is pivotal for tissue homeostasis and repair that the initial inflammatory immune response is followed by an active phase of resolution of inflammation and scar tissue. Recently, it has been shown that after insult, monocyte-derived M2-like macrophages are recruited to the site of injury and that these have an essential role as inflammation-resolving cells in recovery from acute CNS injury. The anti-inflammatory activity displayed by M2-like macrophages, notably their IL10 expression, is required for regulation of the activated microglia (162, 163). In addition, their expression of matrix degrading enzymes favors axonal regrowth by degradation of the glial scar (164). CNS specific T cells facilitate recruitment of blood-borne M2-like macrophages to the CNS through the choroid plexus within the blood–CSF barrier (165). Age-related Th2 inflammation is associated with chronically elevated IL4 levels, which can disrupt choroid plexus barrier functions and thus prevent the resolution of pro-inflammatory processes and induce a state of CNS inflammaging.

## Therapeutic Measures and Future Prospects

**Figure 4:**
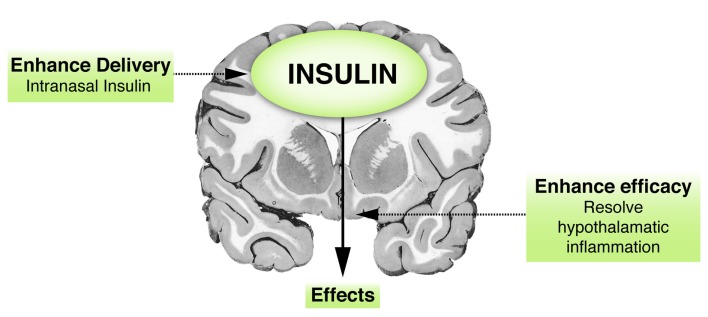
**Insulin and the brain: therapeutic implications**. Hypothetical figure presenting two possibilities of enhancing brain insulin action. First, a way of increasing insulin concentrations in the brain is via enhanced delivery, such as delivery via the intranasal route, which has been shown to have some beneficial effects. Second, insulin action could probably also be augmented by enhancing its efficacy, for example, via resolution of brain (hypothalamic) inflammation.

Since brain insulin has been linked with aging, two possible mechanisms can be proffered for enhancing brain insulin action (Figure 4). Enhanced insulin efficacy might occur through measures aimed at minimizing inflammation; and enhanced delivery might be promoted to the brain areas that are crucial for healthy longevity.

Inflammation, including that occurring in the hypothalamus, has been linked to age-related decline in insulin sensitivity. It has been shown that hypothalamic microglia hyperactivation is regulated by metabolic hormones [leptin, glucagon-like peptide 1 (GLP-1)] and diet but not by body weight *per se* (166). Inflammaging may be treatable and preventable through changes in lifestyle. Interventions that are currently applied to reduce the state of low-grade chronic inflammaging include low dosing of aspirin or statins, weight loss, and exercise. Notably, a lower intake of calories and food that is rich in saturated fat and carbohydrates has been shown to reduce inflammaging (167). In mice, it was shown that hypothalamic inflammation can be resolved by central administration of omega3 and omega9 fatty acids after which body weight regulation and food intake were normalized (168). Physical exercise is known to be protective against numerous diseases and reduction of inflammation has been implicated in the health benefits conferred by exercise (169). Recently, in mice, exercise has also been shown to protect against hypothalamic inflammation induced by high-fat diet (170). Future research may focus on hypothalamic microglia as relevant targets for prevention and treatment of metabolic disorders.

The strong blood glucose lowering effects of intravenously administered insulin have hampered research on the role of insulin in the brain. These hypoglycemic effects can be circumvented by intranasal administration of insulin, which is an innovative way to enhance insulin concentration in the brain without affecting insulin concentration in the circulation (171). Intranasal administration of insulin was shown to be safe and effective in numerous studies in healthy humans and in patients with metabolic disease or cognitive impairment (172). Sub-chronic intranasal insulin application in humans was shown to decrease food intake and weight gain (92) in healthy young men, and to improve declarative memory and mood (173). Moreover, sub-chronic intranasal insulin application in humans was also shown to decrease HPA activation in response to a social stress test. It was shown that insulin may also influence meal-induced thermogenesis and postprandial insulin levels (174). Future research may focus on unraveling the effects of intranasal insulin on other aspects of energy and glucose metabolism in different age groups.

## Conclusion

Insulin is the most powerful anabolic hormone discovered to date. Besides the well-established action of insulin in peripheral organs, such as liver, muscle, and adipose tissue, it is becoming increasingly clear that insulin affects important features of glucose metabolism via central mechanisms. Insulin signaling has been linked to longevity in organisms ranging from nematodes to mammals. While insulin is clearly a neuropeptide in nematodes, it is not yet clear how central insulin contributes to the differences in glucose metabolism that are observed in the context of conditions that are associated with accelerated aging, such as obesity, and delayed aging, such as healthy human longevity. However, novel data indicate that obesity is associated with reduced brain insulin action. Potential mechanisms that contribute to deficits in brain insulin action are impaired transport of insulin from the periphery to the brain and reduced brain insulin sensitivity due to hypothalamic inflammation. In contrast, we speculate that healthy longevity is associated with preserved brain insulin action, and discuss potential ways of enhancing brain insulin action in old age. Given the increasing prevalence of population aging, improving brain insulin action may represent an important therapeutic option to facilitate health in old age.

## Conflict of Interest Statement

The authors declare that the research was conducted in the absence of any commercial or financial relationships that could be construed as a potential conflict of interest.
